# The double burden of malnutrition: rethinking clinical and policy responses in the era of rising obesity in low- and middle-income countries

**DOI:** 10.1017/S1368980026101840

**Published:** 2026-01-13

**Authors:** Akim Tafadzwa Lukwa, Denis Okova

**Affiliations:** 1 Division of Family Medicine, Family, Community and Emergency Care (FaCE), Faculty of Health Sciences, University of Cape Town, Cape Town, South Africa; 2 Health Economics Unit, School of Public Health, University of Cape Town, Cape Town, South Africa

**Keywords:** Double burden of malnutrition, Low- and middle-income countries, Food systems and equity, Primary health care integration, Double-duty actions for nutrition

## Abstract

**Objective:**

To discuss the growing challenge of the double burden of malnutrition (DBM), the co-existence of undernutrition and obesity, and the associated clinical and policy complexities in low- and middle-income countries (LMIC).

**Design:**

This commentary synthesises evidence from recent multi-country and country-specific studies in sub-Saharan Africa and other LMIC. Many LMIC are typified by food insecurity, socio-economic inequalities and fragile health systems which drive DBM patterns, as well as informal community structures such as rotating savings groups which influence access to healthier diets.

**Results:**

Evidence indicates that DBM disproportionately affects disadvantaged households and complicates obesity management. Current clinical guidelines remain obesity-centric and often overlook contexts where individuals with obesity may also experience stunting or micronutrient deficiencies.

**Conclusions:**

This commentary aligns with global frameworks including WHO’s double-duty actions for nutrition, the UN Decade of Action on Nutrition (2016–2025) and the FAO-WFP food systems agenda. To achieve health equity, a coordinated approach is needed: clinical practice must improve diagnosis of co-existing undernutrition and obesity, while public policy must ensure that efforts to manage obesity are supported by food systems that provide equitable access to affordable, nutritious diets.

## Graphical abstract

**Figure:**
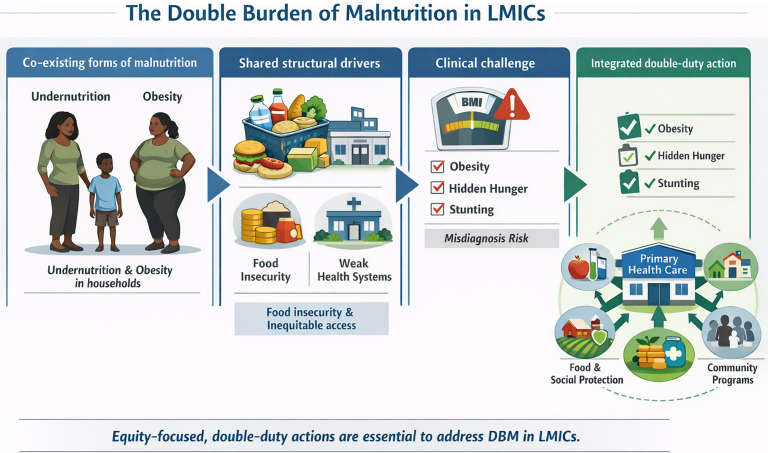

The global nutrition landscape has changed dramatically over the past three decades. While undernutrition manifesting as stunting, wasting, and micronutrient deficiencies remains a critical challenge, overweight and obesity are increasing at unprecedented rates, even in countries still grappling with poverty and food insecurity^(1)^. This paradox, known as the double burden of malnutrition (DBM), represents one of the most complex clinical and policy challenges of the 21st century^(2)^. Recognising this, the WHO, FAO and World Food Programme (WFP) have positioned DBM at the centre of global nutrition action. The UN Decade of Action on Nutrition (2016–2025) calls for integrated, ‘double-duty’ actions that address both undernutrition and obesity through coherent food, health and social protection systems^(3,4)^.

The Lancet Commission on the Global Syndemic of Obesity, Undernutrition and Climate Change identifies these as interlinked crises driven by shared structural determinants such as food system inequities and policy inertia^(5)^. Likewise, the EAT–Lancet Commission on Food in the Anthropocene underscores the need for a ‘Great Food Transformation’ that delivers sustainable, nutrient-rich diets within planetary boundaries^(6)^. In sub-Saharan Africa, DBM has evolved from a household phenomenon into a structural problem affecting entire populations. Recent analyses demonstrate that DBM disproportionately impacts poorer households, with undernutrition persisting among children while overweight and obesity rise among adults, particularly women^(7,8)^. In short, this commentary argues that solving the DBM requires a dual approach: clinicians must learn to treat patients for both obesity and hidden hunger at the same time, while policymakers must ensure that everyone, especially the poor, has access to affordable, healthy food. The best place to start connecting these efforts is within local health clinics.

## Clinical complexity of double burden of malnutrition

### Double burden of malnutrition as a diagnostic challenge of overlapping malnutrition

Clinicians in low- and middle-income countries (LMIC) increasingly encounter patients presenting with overlapping nutritional conditions. Traditional indicators such as BMI are insufficient for detecting DBM, as they fail to reveal underlying stunting, wasting, or micronutrient deficiencies [*We acknowledge that micronutrient deficiency is part of the Triple Burden framework. Here, we treat it as a critical component that complicates both undernutrition and overnutrition, which is a common clinical reality in DBM*] (often called ‘hidden hunger’)^(2,8)^. The WHO Commission on Ending Childhood Obesity warns that standard growth measures, such as ‘BMI-for-age’ (which assesses if a child’s weight is appropriate for their age)^(3)^, can be misleading. They may fail to identify the early onset of excess fat storage in childhood, which signals a hidden risk for developing conditions like type 2 diabetes and heart disease later in life. This is common in children whose growth has been stunted, who may appear to have a normal weight-for-height (*A measure of wasting or acute undernutrition*) but are metabolically unhealthy. Similarly, adults with obesity often consume calorie-dense but nutrient-poor diets, leading to the simultaneous condition of being overweight while deficient in essential vitamins and minerals^(8)^.

### Double burden of malnutrition and clinical outcomes

The co-existence of obesity with undernutrition increases the risk of poor clinical outcomes across the life course. Stunted but overweight children have heightened susceptibility to insulin resistance, metabolic syndrome and early-onset type 2 diabetes^(9,10)^. Evidence from Asia and Latin America shows similar trends, where micronutrient-poor diets and early growth restriction compound obesity-related co-morbidities in adulthood^(5)^. Among women, obesity coupled with iron and folate deficiency elevates risks of pre-eclampsia, gestational diabetes and adverse birth outcomes^(3)^.

### Clinical management dilemmas

Existing clinical frameworks are poorly suited to the complex nutritional realities in LMIC. Weight-loss interventions focused solely on caloric restriction may aggravate deficiencies, while undernutrition programmes promoting energy supplementation without attention to diet quality risk worsening obesity^(4)^. The EAT–Lancet Commission advocates for dietary quality assessment within clinical practice, emphasising nutrient adequacy and food diversity as essential components of both obesity care and undernutrition management^(6)^.

### Lessons from recent evidence

Cross-country analyses confirm the heterogeneity of DBM manifestations. Studies from sub-Saharan Africa, India and Mexico reveal simultaneous rises in childhood stunting and adult obesity, concentrated among lower socio-economic groups^(7,11)^. This reflects the nutrition transition driven by urbanisation and processed food consumption, as described by the Global Food Policy Report^(11)^. Together, these findings underscore that DBM is not simply a statistical co-existence but a dynamic metabolic and social phenomenon requiring integrated diagnostic and treatment approaches.

## Health systems and inequality dimension

### Double burden of malnutrition as an equity issue

DBM is increasingly recognised as a marker of structural inequality within and across LMIC. Evidence shows that DBM is pro-poor, with disadvantaged populations bearing a disproportionate burden^(2,8)^. Rural households, those with limited education, and the lowest income quintiles are most affected. In Zimbabwe, for example, inequalities in child malnutrition worsened between 2010 and 2015, driven by persistent food insecurity and urban–rural disparities^(12)^. Globally, WHO estimates that 462 million adults are underweight, while 1·9 billion are overweight or obese, and 41 million children under five are overweight^(4)^. These patterns reflect shared structural drivers such as poverty, dietary transition and weak food systems that create overlapping vulnerabilities across generations.

### COVID-19 and shocks to food security

The COVID-19 pandemic further revealed how fragile food and health systems are in LMIC. In South Africa, lockdowns intensified hunger, with the poorest households most affected^(13)^. These disruptions worsened DBM, leading to concurrent stunting, wasting and overweight among children^(14–16)^. The pandemic also constrained essential primary health care (PHC) services such as growth monitoring, antenatal care and nutrition counselling reducing the system’s capacity to prevent malnutrition in all its forms^(17)^. WHO has underscored the need to embed nutrition action within resilient, community-oriented PHC systems that can respond to shocks while maintaining continuity of care.

### Implications for health systems

Siloed interventions that separately target undernutrition or obesity are increasingly ineffective. Conventional obesity programmes, often modelled on high-income settings, assume stable access to nutrient-rich foods, an assumption that fails in resource-limited contexts. The WHO Primary Health Care Operational Framework and the UN Decade of Action on Nutrition (2016–2025) advocate for double-duty actions and integrated equity-driven interventions addressing both undernutrition and obesity through shared PHC delivery platforms^(18)^. These include routine nutrition screening at PHC facilities, integration of maternal and child nutrition services, and coordination with agricultural and social protection sectors. Without such systemic reform, LMIC risk perpetuating intergenerational malnutrition and health inequity. Strengthening PHC as a coordinating hub for multi-sectoral nutrition action is thus central to achieving equitable progress towards the Sustainable Development Goals.

## Clinical and policy complexities, and recommendations to address them

The DBM presents a fundamental mismatch between complex, co-existing clinical realities and siloed, single-focus policy responses. This section outlines the core challenges and proposes integrated, ‘double-duty’ recommendations aligned with global frameworks to address them.

### Complexity: fragmented clinical guidelines and diagnostic oversight

Clinicians in LMIC face the challenge of managing patients with overlapping conditions, such as obesity concurrent with stunting or micronutrient deficiencies, yet guidelines and training often address undernutrition and overnutrition separately^(8)^. Reliance on simplistic indicators like BMI fails to capture this complexity, leading to misdiagnosis and ineffective care, such as weight loss advice that exacerbates deficiencies or nutritional supplementation that worsens obesity^(4)^.

### Recommendation: integrate nutrition screening into primary health care

To address this, clinical protocols must be revised to mandate routine screening for the full spectrum of malnutrition within PHC. This includes assessing dietary diversity, micronutrient status and stunting in all patients, especially those presenting with obesity^(3,4)^. This ensures that care for one condition does not inadvertently worsen another, moving clinical practice beyond BMI-based management to a more holistic, person-centred approach.

### Complexity: Siloed national policies and programmes

Despite global consensus on their shared drivers such as poverty, inequitable food systems and weak PHC, national nutrition policies in LMIC remain starkly fragmented, with separate programmes for undernutrition and obesity^(19)^. This policy inertia is often compounded by commercial interests that promote unhealthy, ultra-processed foods^(5)^.

### Recommendation: implement national double- and triple-duty actions

Policymakers must explicitly adopt and fund integrated policies that simultaneously address undernutrition, obesity and related non-communicable diseases^(4,5)^. Evidence-based actions include:

- Fiscal policies: Implementing taxes on sugar-sweetened beverages and subsidies for fruits, vegetables and other nutrient-dense foods^(5)^.
- Food system reform: Aligning agricultural, trade and health sectors to increase the supply and affordability of healthy foods^(6)^.
- Updated programmes: Redesigning school feeding and maternal nutrition schemes to ensure they provide both adequate calories and high nutrient density, combating both stunting and obesity risks^(4,18)^.

### Complexity: systemic inequality and exclusion of community assets

DBM disproportionately affects the poorest households, yet policies often fail to address this socio-economic gradient^(7)^. Furthermore, top-down approaches frequently overlook existing community structures, such as Rotating Savings and Credit Associations (ROSCAs) and stokvels, which possess untapped potential to enhance food security and social capital^(20,21)^.

### Recommendation: leverage community platforms and strengthen social protection

Interventions must be explicitly designed for equity. This involves:

- Strengthening Social Protection: Building resilient safety nets to buffer families from economic shocks, climate crises and pandemics that exacerbate DBM^(7,22,23)^.
- Embedding Nutrition in Community Finance: Integrating nutrition education and behaviour-change support into existing community savings groups to channel pooled resources towards nutrient-rich, rather than just energy-dense foods^(20,21)^.
- Targeting Vulnerable Groups: Ensuring that PHC-linked interventions, such as antenatal nutrition counselling and breastfeeding support, proactively reach the most disadvantaged populations^(3)^.

## Conclusion: a call for coherent action

Tackling the DBM demands a decisive break from policy silos and clinical reductionism. The path forward requires a coherent strategy that links enhanced clinical diagnosis with food system policies and equity-focused social protection. For clinicians, this means adopting integrated screening, for policymakers, it means implementing double-duty actions, and for researchers, it means generating evidence on what works in real-world LMIC settings. Embedding these responses within the frameworks of Universal Health Coverage and the Sustainable Development Goals particularly SDGs 2, 3 and 10 is not just strategic but essential to achieving healthier, more equitable societies.
